# Shape-Sensing Robotic Bronchoscopy with Integrated Mobile Cone-Beam CT Guidance for Intraoperative Localization of Lung Tumors Using Indocyanine Green

**DOI:** 10.3390/diagnostics16121893

**Published:** 2026-06-18

**Authors:** Abdul Rahman Halawa, Miguel Belmonte, Kyle G. Mitchell, Mara B. Antonoff, Ravi Rajaram, Stephen Swisher, David C. Rice, Roberto F. Casal

**Affiliations:** 1 Department of Pulmonary and Critical Care Medicine, McGovern School of Medicine, Houston, TX 77030, USA; 2 Texas Tech School of Medicine, Lubbock, TX 79430, USA; miguel.belmonte@ttuhsc.edu; 3 Department of Cardiothoracic Surgery, The University of Texas MD Anderson Cancer Center, Houston, TX 77030, USA; 4 Department of Pulmonary Medicine, The University of Texas MD Anderson Cancer Center, Houston, TX 77030, USA

**Keywords:** bronchoscopy, indocyanine green, cone-beam computed tomography

## Abstract

**Background/Objectives:** With increasing frequency in sublobar resections, accurate intraoperative localization has become essential to ensure adequate resection margins and spare lung parenchyma. Our study evaluates the efficacy of shape-sensing robotic bronchoscopy (SS-RAB) with integrated mobile cone-beam CT (mCBCT) for intraoperative localization of lung tumors using indocyanine green (ICG). We further aimed to explore the feasibility of a single intubation-single positioning technique for bronchoscopy and surgery. **Methods:** We retrospectively reviewed patients who underwent SS-RAB with integrated mCBCT for ICG marking, followed by minimally invasive sublobar resection. ICG marking was deemed successful when it allowed the operative team to localize and resect the lesion with adequate pathology margins. **Results:** A total of 28 patients with 30 pulmonary lesions from a single institution were included. Median tumor size was 10.5 mm (IQR, 8.7–14.6 mm) and distance from pleura 7.8 mm (IQR, 2.45–13.8 mm). Twenty lesions (66.6%) were solid, 5 lesions (16.6%) semi-solid, and 5 lesions (16.6%) ground-glass. ICG localization was successful in 28 lesions (93%). Nineteen patients (68%) were intubated only with a double-lumen endotracheal tube (DL-ETT), used for bronchoscopy and surgery, and in 10 patients (36%) ICG marking and surgery were both performed in lateral decubitus. One patient developed a small pneumothorax during bronchoscopy which did not prevent ICG injection. **Conclusions:** SS-RAB with integrated mCBCT for ICG marking is successful and safe. Single intubation with DL-ETT and lateral decubitus positioning for both bronchoscopy and surgery are feasible. Further studies are needed to prove a potential increase in efficiency with this technique.

## 1. Introduction

The increasing detection of small, sub-solid, or ground-glass opacity (GGO) lung nodules driven by lung cancer screening initiatives, coronary artery calcium scans, and the wide-spread use of chest computed tomography (CT), may pose challenges for intraoperative localization at the time of resection by thoracic surgeons [1,2]. While lobectomy has been the gold-standard treatment for early-stage non-small-cell lung carcinoma (NSCLC) in patients with adequate pulmonary function, sublobar resection (segmentectomy, wedge resection) has become an established choice of treatment in those with limited functioning, advanced age, and important comorbidities. Its role can also be diagnostic, notably in the case of sub-centimeter lesions and ground-glass opacities (GGOs). Moreover, a recent large randomized controlled non-inferiority trial (CALGB 140503) has proven that sublobar resections are not inferior to lobar resections for peripheral lung cancers of up to 2 cm [3]. With this, sublobar resections may soon become the standard of care in this population as well. Unfortunately, small or subsolid lung nodules, especially if they are not near the pleura, are difficult to localize intraoperatively during minimally invasive surgery such as video-assisted thoracoscopic surgery (VATS) or robot-assisted thoracoscopic surgery (RATS). Such challenges arise from lack of direct tactile feedback and palpation and limited visual distortion of the surface of the lung. Accurate localization is essential to ensure adequate resection margins, avoid unnecessary lung tissue removal, and reduce conversion to thoracotomy, or conversion from sublobar resection to lobectomy for diagnostic or therapeutic purposes.

Previously established localization techniques include percutaneous approaches using hook-wires, microcoils, fiducials, or dyes (such as methylene blue and indocyanine green [ICG]) under CT guidance, as well as bronchoscopic approaches. While percutaneous techniques carry risks such as pneumothorax, marker dislodgement, embolization, coil migration and pulmonary hemorrhage, bronchoscopic localization techniques, predominantly performed via electromagnetic navigational bronchoscopy (ENB), have been historically less accurate [2,4,5,6]. The advent of shape-sensing robotic-assisted bronchoscopy (SS-RAB) and mobile cone beam CT (mCBCT) guidance has recently revolutionized the field of peripheral bronchoscopy [7,8,9,10,11]. The integration of these technologies allows bronchoscopists to reach small targets virtually anywhere in the lungs, safely achieving access to lesions with excellent diagnostic yield [7,8,9,10,11]. The use of SS-RAB with integrated mCBCT for intraoperative localization of lung nodules has not been reported. In this study, we aimed to assess the efficacy and safety of the use of SS-RAB with integrated mCBCT for intraoperative localization of lung nodules utilizing ICG. We also introduce a novel “single-intubation” and “single positioning” strategy, with both robotic bronchoscopy and surgery performed via double-lumen endotracheal tube (DL-ETT) and in lateral decubitus, with hope to improve efficiency and accurately identify nodules in areas prone to atelectasis.

## 2. Materials and Methods

This was a retrospective review of patients who underwent RAB with integrated mCBCT for ICG marking, followed by immediate minimally invasive sublobar resection between February 2024 and October 2025 at the University of Texas MD Anderson Cancer Center. All patients were included in a continuous fashion with no exclusion criteria. Data was extracted from medical records after obtaining ethical approval from the local Institutional Review Board (2025-1126). Extracted data included patient demographics (age, sex, ethnicity, smoking history, ECOG, ASA score, comorbidities), lesion characteristics (size, location, appearance, distance from pleura), procedural details (anesthesia time, localization time, surgical approach, ICG volume, needle gauge, cone-beam CT spins), ICG localization outcomes, surgical pathology margin, and complications (including bronchoscopic, anesthesia, surgical, and post-operative complications). Furthermore, outcomes including length of stay and 30-day mortality were evaluated.

Procedural times were extracted from anesthesia records and defined as follows: “anesthesia time” was the interval from intubation to extubation, encompassing the entire procedure; “bronchoscopy and preparation for surgery time” was the interval from procedure start (initiation of robotic bronchoscopy) to incision time (start of surgical resection); and “surgery time” was the interval from incision time to procedure end (skin closure). ICG marking was deemed successful when ICG was found within the target or in the desired injection site (i.e., between the target and the pleura), as assessed by the operating surgeon, allowing for successful intraoperative localization of the tumor and subsequent resection with grossly adequate pathology margins.

### 2.1. Procedures

**Figure 1:**
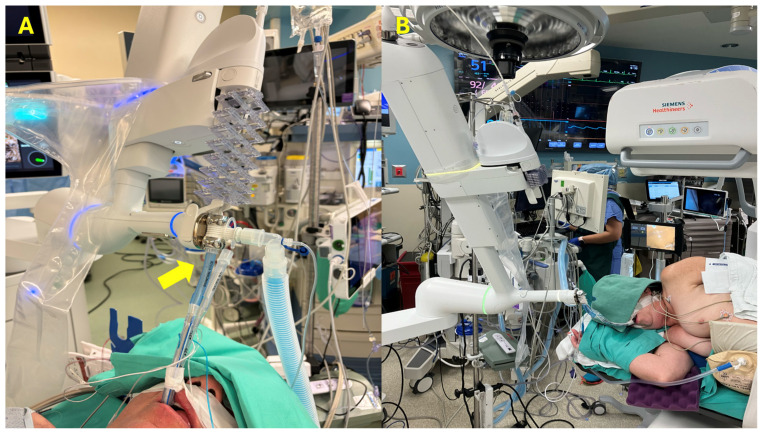
Use of double-lumen endotracheal tube and lateral decubitus for bronchoscopy and surgery. (**A**). The bronchial limb of the double lumen endotracheal tube (arrow) positioned in the trachea is utilized for robotic bronchoscopy and ventilation, while the tracheal limb is left open with its cuff down. (**B**). Patient positioned in lateral decubitus for bronchoscopy and surgery.

ICG marking was performed in the operating room (OR) immediately before the thoracic surgery, during the same anesthesia event. In patients with lesions located posteriorly in dependent areas at high risk for atelectasis (which can obscure the targets on CBCT), both robotic bronchoscopy with ICG marking and surgical resection were performed in lateral decubitus (target side up), with only one intubation with a double-lumen endotracheal tube (DL-ETT) (Figure 1) [12,13,14,15]. In these cases, the tip of the bronchial lumen of the DL-ETT was positioned above the carina in the distal trachea for the robotic bronchoscopy/ICG marking portion of the procedure. The bronchial lumen cuff was inflated, and the bronchial lumen of the DL-ETT was utilized both for ventilation and robotic bronchoscopy, leaving the tracheal lumen cuff deflated, with its proximal end disconnected from the ventilator (open). The swivel adaptor for the bronchoscopy robot was connected to the proximal end of the bronchial lumen of the DL-ETT, allowing for ventilation and robotic bronchoscopy through the bronchial lumen. Once the ICG marking was over, the DL-ETT was advanced over a bronchoscope into the left main stem bronchus and was then utilized in a traditional fashion for the surgical resection. In patients with lesions in non-dependent areas, robotic bronchoscopy and ICG marking were performed in a supine position. These patients were either intubated with a DL-ETT for both procedures (bronchoscopy and surgery, as described above), or were intubated with a single-lumen endotracheal tube (SL-ETT), and, after ICG marking, they were then extubated and re-intubated with a DL-ETT and placed in lateral decubitus for surgery.

**Figure 2:**
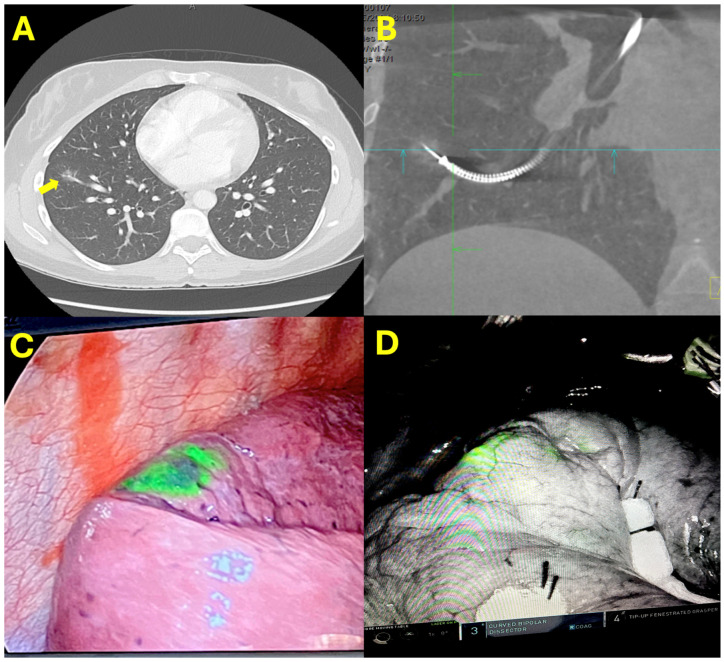
Indocyanine green localization. (**A**). Right lower lobe ground glass lesion (arrow). (**B**). Mobile-cone beam CT showing needle in target prior to injection. (**C**). Indocyanine green view during video-assisted thoracic surgery. (**D**). Indocyanine green view during robotic-assisted thoracic surgery.

Shape-sensing robotic-assisted bronchoscopy (SS-RAB) using the Ion Endoluminal System, Intuitive Surgical Inc., Sunnyvale, CA, USA, was performed with mobile cone-beam CT (mCBCT) guidance (Cios 3D Spin mobile, Siemens Healthineers, Erlangen, Germany). “Integration” software (6th generation) which allows for automatic transfer of image data from the mCBCT to the robotic platform to update the virtual target location was utilized in all cases [9]. After standard registration and navigation towards the virtual target, a first mCBCT spin was performed for integration purposes. During the integration process, the desired localization of the injection was discussed between the bronchoscopist and the surgeon, and it was selected as the “updated” target location. The ICG injection site could be within the actual target if it was close enough to the pleura for ICG detection (up to 1.5 cm from pleura), or somewhere between the target and the pleural surface, when targets were too deep. After updating the target, utilizing the near and far target distances provided by the robotic platform, the needle was deployed at the desired depth. A repeat mCBCT spin was performed to evaluate the position of the needle. If this was not satisfactory, the needle was drawn back, adjustments were made based on these new images, and the needle was once again deployed. Once the needle was at the desired position, ICG injection was performed with a 21G needle (Flexision Needle, Intuitive Surgical). Twenty-five miligrams of lyophilized ICG powder were diluted in 10 mL of sterile water. From 0.5 to 1 mL of this ICG preparation was injected. After injection, the robotic bronchoscope was withdrawn, and after either advancing the DL-ETT or replacing the SL-ETT for a DL-ETT, we proceeded with VATS or RATS sublobar resection (wedge, or segmentectomy). Intraoperatively, ICG was detected with near-infrared imaging (NIR) (Firefly, Intuitive Surgical Inc.) during RATS, or with a NIR thoracoscope (1788 Platform, Stryker, Portage, MI, USA) during VATS (Figure 2). Resection margins were confirmed intraoperatively by frozen section when indicated at surgeon discretion.

### 2.2. Statistical Analysis

Continuous variables were reported as median with interquartile range (IQR), and categorical variables as frequencies and percentages. Statistical analysis was performed using SAS version 9.4.

## 3. Results

A total of 28 patients with 30 pulmonary lesions underwent shape-sensing robotic-assisted bronchoscopy with integrated mCBCT guidance and ICG dye marking followed by immediate resection. The median age was 61 years (IQR, 52.8–67.5 years), and 57% (16/28) were female. Fourteen patients (50%) had primary lung cancer, 13 patients (46.4%) had metastatic disease (two of these patients had two lesions resected), and one patient was found to have atypical adenomatous hyperplasia. Pathologic diagnosis was obtained prior to surgery in 19 patients (67.8%) −15 of those diagnosed via SS-RAB and four with CT-guided percutaneous biopsy. Lesions (*n* = 30) had a median long axis of 10.5 mm (IQR, 8.7–14.6 mm) and distance from the pleura of 7.8 mm (IQR, 2.45–13.8 mm). Twenty lesions (66.6%) were solid, 5 lesions (16.6%) were semi-solid, and 5 lesions (16.6%) were purely ground-glass. The median bronchial generation reached was 9 (IQR, 8–10), with a bronchus sign present in only two cases (7%). Further details on baseline patient and nodule characteristics are depicted in Table 1 and Table 2, respectively.

ICG localization was successful in 28 lesions (93%). In two lesions (7%), ICG was found outside the desired area, but the target was still identified intraoperatively. Final pathology margins were negative in all 30 lesions. Nineteen patients (68%) were intubated only once with a DL-ETT for both ICG marking and surgery, and nine (32%) were initially intubated with a SL-ETT for ICG marking which was subsequently replaced by a DL-ETT for surgery. Eleven patients (39%) had lesions located in dependent areas and both ICG marking and surgery were performed in lateral decubitus. Median anesthesia time was 203 min (IQR, 176–238 min), bronchoscopy and preparation for surgery time was 58 min (IQR, 47–71 min), and surgery time was 99 min (IQR, 81–128 min). Median bronchoscopy and preparation for surgery time in patients who underwent only one intubation with DL-ETT and were positioned in lateral decubitus for both procedures was 47 min (IQR, 45–69.5), and it was 59 min (IQR, 55–75) for those who were subject to two intubations and had bronchoscopy in supine position (*p* = 0.215). The surgical approach was RATS wedge resection in 20 lesions (66.7%), VATS wedge resection in 8 lesions (26.7%), and RATS segmentectomy in 1 lesion (3.3%). One patient converted from RATS to open thoracotomy with wedge resection due to the presence of adhesions.

One patient developed a small pneumothorax (<30%) during bronchoscopy immediately before ICG injection. The pneumothorax was detected with mCBCT, the needle location was adjusted, and ICG injection was subsequently performed, leading to a successful intraoperative localization. There was no extravasation of ICG in the pleural space. There were no other complications related to anesthesia or robotic bronchoscopy. Post-operative complications were limited to persistent air leak (>5 days) in two patients (7%), and a new home oxygen requirement in one patient with known chronic obstructive pulmonary disease (COPD) and pre-operative moderate airway obstruction and DLCO of 60%. Median length of stay was 3 days (IQR, 2–4 days), with no 30-day mortality. Further procedural details and surgical outcomes are described in Table 2 and Table 3, respectively.

## 4. Discussion

This study demonstrates a highly successful surgical localization rate with a favorable safety profile and describes a novel approach of a single intubation with DL-ETT and single positioning—lateral decubitus—for both robotic bronchoscopy and surgery. To the best of our knowledge, this is the first report of the use of SS-RAB with integrated mCBCT for intraoperative localization of lung tumors with ICG injection.

The increasing detection of small, subsolid, and ground-glass pulmonary nodules has increased the demand for precise intraoperative localization, to facilitate minimally invasive and lung-sparing resection. An ideal method for intraoperative localization would be one that is highly accurate regardless of the type and location of the lung nodule, painless and safe for the patient, and maximizes efficiency and aligns with surgical workflow (i.e., single setting along with surgery) [2]. Historically, percutaneous approaches for intraoperative localization have shown higher accuracy than bronchoscopy, but with increased complication rates and more complex logistics. The most widely utilized percutaneous approach is the use of hook-wire. While its localization success rate ranges between 94 and 98%, it is unfortunately associated with high rates of pneumothorax (35–45%), patient discomfort, pulmonary hemorrhage (16%), and potential dislodgement (2–4%) [2,6,16]. Utilizing microcoils instead of hook-wire has been shown to have comparable accuracy but with lower rate of pneumothorax and bleeding, becoming a promising percutaneous alternative [17,18]. ICG has emerged as a preferred fluorescent dye for marking. It is an amphiphilic dye that is non-toxic and has a peak wavelength of nearly 830 nm under near-infrared light, offering deep tissue penetration (up to 2.0 cm) and visualization under NIR thoracoscopy [19,20]. Unlike methylene blue, it does not need to be injected immediately below the pleura to be detected. Percutaneous injection of ICG has been reported, but the development of pneumothorax with the percutaneous approach can prevent successful marking in up to 20% of the patients [19].

Bronchoscopists have struggled to reach small peripheral lung lesions for many decades. The combination of fluoroscopy, radial-probe endobronchial ultrasound (RP-EBUS), and electromagnetic navigation technologies were simply not able to surpass a diagnostic yield of around 50–70% [21,22]. False positive RP-EBUS due to atelectasis, CT to body divergence (difference in shape and volume of the lungs during the pre-procedural diagnostic CT scan utilized for planning and the shape and volume of the lungs while under anesthesia), and the lack of an accurate method to confirm target reach were among the most important barriers of these techniques [14,23,24]. The recent advent of SS-RAB and CBCT guidance has had an enormous impact in the field of bronchoscopy. SS-RAB provides maneuverability, reach, and stability, allowing the bronchoscopist to target nodules virtually anywhere in the lungs. The addition of CBCT imaging to SS-RAB allows for correction of CT to body divergence, proper confirmation of tool in lesion, and avoidance of injury to pleura or vital structures. While the correction for CT to body divergence initially required the proceduralist’s interpretation of the intraoperative CBCT images, it is now seamlessly performed by the “integration” software of the robotic platform which analyzes the newly obtained CBCT images and updates the location of the virtual target during bronchoscopy, reducing mental burden, procedural time, and number of CBCT spins [9]. The combination of these technologies results in a nearly 100% rate of tool in lesion with a <1% of pneumothorax rate, hence the interest in its use for intraoperative localization of lung tumors [7,8,9,10,11,25].

The use of SS-RAB alone (without CBCT image guidance) for intraoperative tumor localization with ICG has been described by Shahoud and coworkers [26]. They included 30 patients with a single nodule each, who underwent SS-RAB with ICG injection immediately before minimally invasive surgery. Injections were performed at the site of the virtual target provided by SS-RAB, without CBCT guidance. The median lesion size was 9 mm (4 to 25 mm), and the median distance from the pleura was 5 mm (1 to 32 mm). Eight lesions were classified as solid (26.7%), 21 lesions were mixed (70%), and 1 was a pure GGO (3.3%). Fifteen patients underwent robot-assisted thoracic surgery (50%), 13 patients underwent uniportal VATS (43.3%), and 2 patients underwent multiportal VATS (6.7%). All patients underwent a sublobar resection (wedge in 26 patients and segmentectomy in 4). The ICG localization success rate was 83.3% (25 of 30). In three cases ICG was not visualized, and in two cases there was free extravasation of dye. They did not experience any complications with regard to SS-RAB ICG injection, two patients (6.7%) had persistent air-leak, and the mean hospital length of stay was 2.3 days. This series is highly similar to the current study in terms of target characteristics (small and peripheral) and procedural outcomes, except for a slightly lower localization success rate. It is not surprising that in the absence of CBCT guidance their intraoperative localization accuracy was acceptable, since a prior study of our group demonstrated that if we deploy a needle at the site of the virtual target provided by SS-RAB (without correcting for CT to body divergence), the needle will most of the time land within 5 mm of the actual target [7]. Of course, CT to body divergence will vary according to the anatomic location of the lesion (being worse in lower lobes) and the degree of atelectasis. Unfortunately, Shahoud and coworkers did not describe the anatomic location of the resected nodules. The use of a different type of robotic bronchoscopy, electromagnetic-guided robotic-assisted bronchoscopy (EMN-RAB), also combined with CBCT, has been reported by Chan and coworkers [27]. In a small series of five patients, they describe a localization success rate of 80%, with the use of a “triple dye”, containing equal volumes of indocyanine green (ICG), methylene blue and iodinated contrast. The mean size of nodules was 8.3 mm (range, 3.5–14 mm), 4 out of 5 nodules were GGOs, and the mean distance from the pleura was at a mean of 9.3 mm. The inability to visualize ICG in 1 out of their 5 cases was attributed to the smaller ICG volume that was injected in that case (0.2 mL). Data preceding the advent of robotic bronchoscopy, with the use of electromagnetic navigational bronchoscopy (ENB) alone, also reported successful ICG localization rates of 80–90% [5]. Of note, lesions in these studies were larger and presumably located in areas accessible to ENB (though anatomic lesion location and bronchus sign rates were not reported). On the contrary, our study included smaller lesions, with bronchus sign only present in 2 out of 30 lesions, and with 43% of the lesions located in lower lobes, which are subject to greater CT to body divergence and atelectasis. Based on our prior experience with ENB, the majority of the lesions included in our study would have been outside the reach of traditional ENB.

The possibility of marking lesions at the time of diagnostic bronchoscopy with a marker that will not alter pathology, and that will remain in place until the moment of surgery is of great interest [28,29,30]. Benn and coworkers recently described the use of ICG-soaked fiducial markers (Cook Tornado Coils, Cook Medical LLC) in a retrospective study that included 54 patients from four institutions [29]. Marking was performed with SS-RAB and either mCBCT, fixed CBCT or 2D Fluoroscopy guidance, depending on the institution, at the time of tumor biopsy. Eight patients had surgery the same day of tumor marking, and 46 were discharged home and returned for surgery at a median of 5 days later (range, 2–13 days). ICG was visible in all lesions during surgery, and all tumors were properly identified. Targets were slightly larger than in our study, and more favorably located with bronchus signs reported in the vast majority (81% vs. 2% in our study). Though this approach may have the advantage of minimizing OR time, it may entail more coordination between bronchoscopists and surgeons (potentially delaying treatment), since surgery needs to be scheduled no longer than 13 days from tumor marking. Moreover, if the pre-test probability of malignancy is not high, institutions that do not count with on-site cytology may not be able to adopt this technique, since it could result in marking of lesions that may not require surgery (i.e., infectious nodules).

We would like to highlight certain technical aspects of our workflow which may streamline these combined procedures in the OR, potentially reducing time, risk, and patient discomfort, and preventing atelectasis from obscuring the targets in CBCT. The use of a single intubation with a DL-ETT was slowly adopted and hence not performed in all cases of this study, but it has now become our standard of care. The SS-RAB catheter has an outer diameter of only 3.5 mm, which allows for adequate ventilation through the bronchial lumen while performing robotic bronchoscopy, even with a 35F DL-ETT. The tip of the bronchial lumen of the DL-ETT needs to remain in the distal trachea (for robotic bronchoscopy), and only the bronchial cuff needs to be inflated. Essentially, we utilize the DL-ETT as if it was a SL-ETT, and we only advance it under bronchoscopy guidance after ICG marking, in preparation for surgery. This prevents the need for two intubations with its associated risks (oropharyngeal trauma, aspiration, throat discomfort, etcetera). We perform robotic bronchoscopy in the lateral decubitus (target side up) in patients with lesions located in zones at high risk for atelectasis, as we described in prior studies [12,14,15,24,31]. This lateral decubitus strategy has been shown to be superior to a ventilatory strategy with high PEEP in a randomized controlled trial, preventing atelectasis from obscuring targets in all cases [12]. This strategy is extremely relevant when dealing with smaller and less solid lesions (typical candidates for sublobar resection) that can easily be obscured with minimal amount of atelectasis (when located in dependent areas). Moreover, having the patient already in lateral decubitus and with a DL-ETT facilitates the transition from robotic bronchoscopy to surgery. The median time for bronchoscopy and preparation for surgery in patients who underwent only one intubation with DL-ETT and were positioned in lateral decubitus for both procedures was shorter (47 vs. 59 min), but this difference was not statistically significant since our study was not powered for this specific analysis.

The total radiation dose reported in our cohort which included the use of fluoroscopy as well as mCBCT was 4.53 Gy-cm^2^ (median). This dose is much lower than that of a diagnostic chest CT (30–40 Gy-cm^2^) or a diagnostic peripheral bronchoscopy with CBCT guidance (20–70 Gy-cm^2^) [32,33]. Unfortunately, radiation doses of CT-guided percutaneous procedures are typically quantified using Dose-Length Product (measured in mGy-cm), which precludes an accurate comparison.

Limitations of this study include its retrospective, single-center design, and small sample size. Another major limitation, shared by most studies on this topic, is the subjective definition of success based on the operating surgeon’s assessment. The actual robotic bronchoscopy time required for ICG marking is not known, since the time from bronchoscopy to surgical incision retrieved from the anesthesia records included non-bronchoscopic procedures such as lines, patient positioning, re-intubation (when applicable), draping, surgical preparation, or surgical equipment setup. The true impact on OR efficiency is still unknown and would have to be assessed in a randomized trial. While robotic bronchoscopy with ICG marking can increase OR time, a quick and accurate identification of the lesion by the surgeons may reduce surgical time and offset the additional time employed for ICG marking. It is clear to the authors that the technologies utilized in this study are of high-cost and not widely accessible. A cost-effectiveness analysis and comparison with other techniques are still needed. Nevertheless, these early results suggest that SS-RAB with integrated mCBCT guidance and ICG marking is a safe, effective, and increasingly reproducible method for localizing challenging pulmonary nodules during minimally invasive surgery. These integrated technologies, when available, may have a profound impact on clinical practice.

## 5. Conclusions

The growing number of minimally invasive sublobar resections of lung tumors has increased the demand for accurate intraoperative localization, to facilitate minimally invasive and lung-sparing resections. The current study demonstrates that injection of ICG via a combination of SS-RAB with integrated m-CBCT has the potential of being a highly accurate and safe methodology for localizing challenging pulmonary nodules during minimally invasive surgery. Further studies are needed to determine whether our approach with single intubation and positioning (lateral decubitus) can also improve procedural efficiency.

## Figures and Tables

**Table 1:** Baseline Patient Characteristics.

Baseline Patient Characteristic	*n* = 28
**Age (years)**	61 (52.8–67.5)
**Sex—no. (%)**	
Female	16 (57.1%)
Male	12 (42.9%)
**Ethnicity—no. (%)**	
White, non-Hispanic	20 (71%)
White, Hispanic	3 (11%)
Black	1 (4%)
Asian	4 (14%)
**Smoking status—no. (%)**	
Never	19 (67.8%)
Former	8 (28.6%)
Current	1 (3.6%)
**COPD—no. (%)**	3 (10.7%)
**Coronary artery disease—no. (%)**	7 (28.0%)
**ECOG performance status—no. (%)**	
0	22 (78.6%)
1	6 (21.4%)
**ASA class—no. (%)**	
2	4 (14.3%)
3	24 (85.7%)
**Cancer type—no.** **(%)**	
*Lung primary*	14 (50.0%)
Adenocarcinoma	11 (39.2%)
Small-cell carcinoma	1 (3.6%)
Carcinoid tumor	1 (3.6%)
Neuroendocrine carcinoma	1 (3.6%)
*Metastatic disease*	13 (46.4%)
Colorectal adenocarcinoma	6 (21.4%)
Other	7 (25.0%)
*Atypical Adenomatous Hyperplasia (AAH)*	1 (3.6%)
**Tumor stage (primary lung cancer)—no.** **(%)**	
Stage I	13 (46.4%)
Stage II	1 (3.6%)

**Table 2:** Lung Nodule and Procedural Characteristics.

Baseline Characteristic	*n* = 30
**Lesion size—mm**	
Long axis—median (IQR)	10.5 (8.7–14.6)
Short axis—median (IQR)	8.2 (6.8–9.9)
**Distance from pleura—mm, median (IQR)**	7.8 (2.45–13.8)
**Lesion** **appearance—no.** **(%)**	
Solid	20 (66.7)
Ground-glass	5 (16.7)
Subsolid	5 (16.7)
**Lesion location—no.** **(%)**	
Right upper lobe	8 (26.6)
Right middle lobe	0
Right lower lobe	6 (20)
Left upper lobe	9 (30)
Left lower lobe	7 (23.3)
**Bronchus sign on imaging—no. (%)**	2 (7.1)
**Procedure time (minutes)—median (IQR)**	*n* = 28
Anesthesia	203 (176–238)
Bronchoscopy and surgical preparation	58 (47–71)
Surgery	99 (81–128)
**Patient Position for Robotic Bronchoscopy**	
Supine	17 (61%)
Lateral Decubitus	11 (39%)
**Intubation Strategy**	
One intubation (DL-ETT)	19 (68%)
Two intubations (SL-ETT followed by DL-ETT)	9 (32%)

**Table 3:** Procedural and Surgical Outcomes.

Characteristic	Value
**ICG Localization—no. (%)**	
ICG on target, surgically located	28 (93)
ICG off target area, target still surgically located	2 (7)
ICG off target area, target not located	0
**Surgical approach—no. (%)**	
RATS wedge resection	20 (66.7)
VATS wedge resection	8 (26.7)
RATS segmentectomy	1 (3.3)
Thoracotomy (Wedge)	1 (3.3)
**Surgical Localization—no. (%)**	30 (100)
**Complications—no. (%)**	
Intraoperative Pneumothorax	1 (3.6%)
Persistent air leak (>5 days)	2 (7.1%)
New home oxygen requirement	1 (3.6%)
**Length of stay (days) median (IQR)**	3 (2–4)
ICU admission	0
30-day mortality	0
**Radiation exposure**	
Fluoroscopy time (min)—median (IQR)	1.4 (1.07–1.62)
Reference air kerma (mGy)—median (IQR)	293 (236.7–361.8)
Dose–area product (Gy·cm^2^)—median (IQR)	4.53 (3.63–5.37)

## Data Availability

The data presented in this study are available on request from the corresponding author due to local institutional policies.

## References

[1] Su K.W., Singhal S., Sarkaria I.S. (2021). Intraoperative imaging and localization techniques for part-solid nodules. JTCVS Tech..

[2] Zhang H., Zhang C., Li L., Qi J., Yang G.H., Li Y.Q., Gong C.Q. (2025). Small pulmonary nodule localization techniques in the era of lung cancer screening: A narrative review. Int. J. Surg..

[3] Altorki N., Wang X., Kozono D., Watt C., Landrenau R., Wigle D., Port J., Jones D.R., Conti M., Ashrafi A.S. (2023). Lobar or Sublobar Resection for Peripheral Stage IA Non-Small-Cell Lung Cancer. N. Engl. J. Med..

[4] Bowling M.R., Folch E.E., Khandhar S.J., Arenberg D.A., Awais O., Minnich D.J., Pritchett M.A., Rickman O.B., Sztejman E., Anciano C.J. (2019). Pleural dye marking of lung nodules by electromagnetic navigation bronchoscopy. Clin. Respir. J..

[5] Geraci T.C., Ferrari-Light D., Kent A., Michaud G., Zervos M., Pass H.I., Cerfolio R.J. (2019). Technique, Outcomes with Navigational Bronchoscopy Using Indocyanine Green for Robotic Segmentectomy. Ann. Thorac. Surg..

[6] Kleedehn M., Kim D.H., Lee F.T., Lubner M.G., Robbins J.B., Ziemlewicz T.J., Hinshaw J.L. (2016). Preoperative Pulmonary Nodule Localization: A Comparison of Methylene Blue and Hookwire Techniques. AJR Am. J. Roentgenol..

[7] Bashour S.I., Khan A., Song J., Chintalapani G., Kleinszig G., Sabath B.F., Lin J., Grosu H.B., Jimenez C.A., Eapen G.A. (2024). Improving Shape-Sensing Robotic-Assisted Bronchoscopy Outcomes with Mobile Cone-Beam Computed Tomography Guidance. Diagnostics.

[8] Fernandez-Bussy S., Yu Lee-Mateus A., Barrios-Ruiz A., Valdes-Camacho S., Lin K., Ibrahim M.I., Vaca-Cartagena B.F., Funes-Ferrada R., Reisenauer J., Robertson K.S. (2025). Diagnostic performance of shape-sensing robotic-assisted bronchoscopy for pleural-based and fissure-based pulmonary lesions. Thorax.

[9] Husta B.C., Cheng G.Z., Batra H., Reisenauer J.S., Bartek W.M., Kalchiem-Dekel O., Zouk A., Patel N., Chawla M., Eapen G.A. (2026). Shape-sensing robotic-assisted bronchoscopy with integrated mobile cone-beam CT for small nodules: Results from the prospective multicentre CONFIRM study. Thorax.

[10] Husta B.C., Menon A., Bergemann R., Lin I.H., Schmitz J., Rakocevic R., Nadig T.R., Adusumilli P.S., Beattie J.A., Lee R.P. (2024). The incremental contribution of mobile cone-beam computed tomography to the tool-lesion relationship during shape-sensing robotic-assisted bronchoscopy. ERJ Open Res..

[11] Reisenauer J., Duke J.D., Kern R., Fernandez-Bussy S., Edell E. (2022). Combining Shape-Sensing Robotic Bronchoscopy with Mobile Three-Dimensional Imaging to Verify Tool-in-Lesion and Overcome Divergence: A Pilot Study. Mayo Clin. Proc. Innov. Qual. Outcomes.

[12] Boster J.M., Goertzen M., Sarkiss M., Armas Villalba A.J., Bhandari B.S., Song J., Jimenez C.A., Sabath B.F., Lin J., Grosu H.B. (2026). Superiority of Lateral Decubitus Strategy in Preventing Atelectasis from Obscuring Targets During Robotic Bronchoscopy: Lateral Decubitus Strategy vs Ventilatory Strategy to Prevent Atelectasis Trial. Chest.

[13] Casal B., Casal R.F. (2026). Best practices in shape-sensing robotic bronchoscopy with mobile cone beam computed tomography guidance: How I do it. J. Thorac. Dis..

[14] Khan A., Bashour S., Sabath B., Lin J., Sarkiss M., Song J., Sagar A.S., Shah A., Casal R.F. (2024). Severity of Atelectasis during Bronchoscopy: Descriptions of a New Grading System (Atelectasis
Severity Scoring System—“ASSESS”) and At-Risk-Lung Zones. Diagnostics.

[15] Lin J., Sabath B.F., Sarkiss M., Jimenez C.A., Casal R.F. (2022). Lateral Decubitus Positioning for Mobile CT-guided Robotic Bronchoscopy: A Novel Technique to Prevent Atelectasis. J. Bronchol. Interv. Pulmonol..

[16] Chu S., Wei N., Lu D., Chai J., Liu S., Lv W. (2022). Comparative study of the effect of preoperative hookwire and methylene blue localization techniques on post-operative hospital stay and complications in thoracoscopic pulmonary nodule surgery. BMC Pulm. Med..

[17] Mayo J.R., Clifton J.C., Powell T.I., English J.C., Evans K.G., Yee J., McWilliams A.M., Lam S.C., Finley R.J. (2009). Lung nodules: CT-guided placement of microcoils to direct video-assisted thoracoscopic surgical resection. Radiology.

[18] Park C.H., Han K., Hur J., Lee S.M., Lee J.W., Hwang S.H., Seo J.S., Lee K.H., Kwon W., Kim T.H. (2017). Comparative Effectiveness and Safety of Preoperative Lung Localization for Pulmonary Nodules: A Systematic Review and Meta-analysis. Chest.

[19] Anayama T., Hirohashi K., Miyazaki R., Okada H., Kawamoto N., Yamamoto M., Sato T., Orihashi K. (2018). Near-infrared dye marking for thoracoscopic resection of small-sized pulmonary nodules: Comparison of percutaneous and bronchoscopic injection techniques. J. Cardiothorac. Surg..

[20] Dai B., Yu A., Zhao G., Wang Y., Zhou Y., Ni K. (2024). Advantages and rational application of indocyanine green fluorescence in pulmonary nodule surgery: A narrative review. J. Thorac. Dis..

[21] Folch E.E., Bowling M.R., Pritchett M.A., Murgu S.D., Nead M.A., Flandes J., Krimsky W.S., Mahajan A.K., LeMense G.P., Murillo B.A. (2022). NAVIGATE 24-Month Results: Electromagnetic Navigation Bronchoscopy for Pulmonary Lesions at 37 Centers in Europe and the United States. J. Thorac. Oncol..

[22] Tanner N.T., Yarmus L., Chen A., Wang Memoli J., Mehta H.J., Pastis N.J., Lee H., Jantz M.A., Nietert P.J., Silvestri G.A. (2018). Standard Bronchoscopy with Fluoroscopy vs Thin Bronchoscopy and Radial Endobronchial Ultrasound for Biopsy of Pulmonary Lesions: A Multicenter, Prospective, Randomized Trial. Chest.

[23] Pritchett M.A., Bhadra K., Calcutt M., Folch E. (2020). Virtual or reality: Divergence between preprocedural computed tomography scans and lung anatomy during guided bronchoscopy. J. Thorac. Dis..

[24] Sagar A.S., Sabath B.F., Eapen G.A., Song J., Marcoux M., Sarkiss M., Arain M.H., Grosu H.B., Ost D.E., Jimenez C.A. (2020). Incidence and Location of Atelectasis Developed During Bronchoscopy Under General Anesthesia: The I-LOCATE Trial. Chest.

[25] Fernandez-Bussy S., Valdes-Camacho S., Barrios-Ruiz A., Vaca-Cartagena B.F., Yu Lee-Mateus A., Hazelett B.N., Chadha R.M., Reisenauer J.S., Edell E.S., Kern R.M. (2025). Streamlining Lung Cancer Diagnosis: One Procedure for Multi-Site Biopsy Using Shape-Sensing Robotic-Assisted Bronchoscopy. Respiration.

[26] Shahoud J., Weksler B., Ghosh S., Ganesh A., Fernando H. (2024). Robot-Assisted Bronchoscopy for Identification of Lung Nodules During Minimally Invasive Pulmonary Resection. Innov. Technol. Tech. Cardiothorac. Vasc. Surg..

[27] Chan J.W.Y., Chang A.T.C., Yu P.S.Y., Lau R.W.H., Ng C.S.H. (2022). Robotic Assisted-Bronchoscopy with Cone-Beam CT ICG Dye Marking for Lung Nodule Localization: Experience Beyond USA. Front. Surg..

[28] Bawaadam H., Benn B.S., Colwell E.M., Oka T., Krishna G. (2023). Lung Nodule Marking with ICG Dye-Soaked Coil Facilitates Localization and Delayed Surgical Resection. Ann. Thorac. Surg. Short. Rep..

[29] Benn B.S., Bawaadam H., Colwell E.M., Peterson M.D., Tisol W.B., Niroula A., Jaber W.S., Khullar O.V., Daymude K., Phan C.T. (2025). Indocyanine Green-Soaked Fiducial Markers for Lung Nodules Prior to Thoracic Surgery. Chest Pulm..

[30] Walsh E.J., Bawaadam H., Mammarappallil J.G., Snider J.R., Allsopp W.C., Brodeur F.J., Green A.R., Krishna G., Wojcik B.M. (2026). Lung Tumors Marked Percutaneously with Indocyanine Green Dye-Soaked Embolization Coils: A Visual Beacon for Accurate Intraoperative Localization during Lung-Sparing Surgery. J. Vasc. Interv. Radiol..

[31] Salahuddin M., Sarkiss M., Sagar A.S., Vlahos I., Chang C.H., Shah A., Sabath B.F., Lin J., Song J., Moon T. (2022). Ventilatory Strategy to Prevent Atelectasis During Bronchoscopy Under General Anesthesia: A Multicenter Randomized Controlled Trial (Ventilatory Strategy to Prevent Atelectasis -VESPA- Trial). Chest.

[32] Nickoloff E.L., Lu Z.F., Dutta A.K., So J.C. (2008). Radiation Dose Descriptors: BERT, COD, DAP, and Other Strange Creatures. Radi-ographics.

[33] Wijma I.N., Casal R.F., Cheng G.Z., Einsiedel P.F., Fantin A., Hall D.J., Herth F.J., Ng C.S., Pritchett M.A., Shah P.L. (2024). Radiation Principles, Protection, and Reporting for Interventional Pulmonology: A World Association of Bronchology and Interventional Pulmonology White Paper. Respiration.

